# P-85. Impact of Magnetic Resonance Imaging on Re-Amputation Rates in Diabetic Foot Osteomyelitis

**DOI:** 10.1093/ofid/ofae631.292

**Published:** 2025-01-29

**Authors:** Arthur Chan, Cassandre Charles, Jiachen Xu, Kari A Mergenhagen, Bethany A Wattengel, Ashley O’Leary, Andrew Puckett, Joseph Nasca, Thomas Russo

**Affiliations:** Veterans Affairs Western New York Healthcare System, Buffalo, New York; VA WNY Healthcare System, Buffalo, New York; Veterans Affairs Western New York Healthcare System, Buffalo, New York; VA WNY Healthcare System, Buffalo, New York; VA WNY Healthcare System, Buffalo, New York; D'youville School Of Pharmacy, Buffalo, New York; VA WNY Healthcare System, Buffalo, New York; VA WNY Healthcare System, Buffalo, New York; VA WNY Healthcare System, Buffalo, New York

## Abstract

**Background:**

Diabetic foot infections constitute 20% of hospital admissions and result in 80% of amputations annually. It is noteworthy that diabetes is more prevalent among U.S. veterans compared to the general population due to age, exposure to environmental factors during service, and mental health conditions which can contribute to unhealthy behaviors and increased risk of diabetes. Magnetic resonance imaging (MRI) is considered the imaging modality of choice for evaluating diabetic foot osteomyelitis given its higher sensitivity. A staging MRI, may decrease subsequent amputation rates in diabetics with osteomyelitis.

**Table 1:**
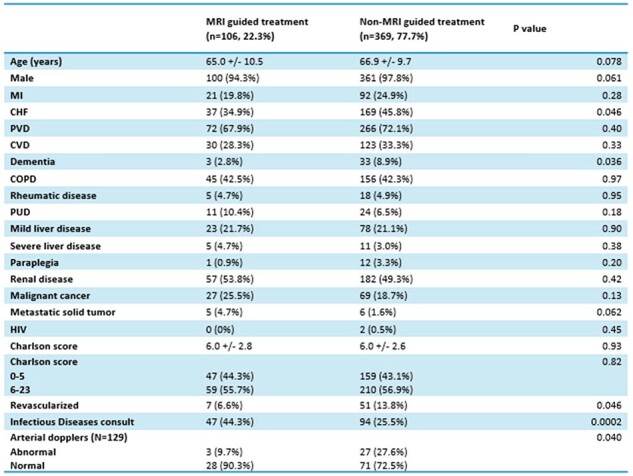
Patient characteristics and comorbidities MRI=magnetic resonance imaging, MI=myocardial infarction, CHF=congestive heart failure, PVD=peripheral vascular disease, CVD=cardiovascular disease, DM=diabetes mellitus, COPD=chronic obstructive pulmonary disease, PUD=peptic ulcer disease, HIV=human immunodeficiency virus

**Methods:**

A retrospective chart review was conducted to analyze the risk of re-amputation in patients with diabetic foot osteomyelitis hospitalized between 2005 and 2022. Patients with and without MRI guided treatment were compared. The primary outcome was the rate of re-amputation at specific time intervals: 30-day, 60-day, 180-day, and 365-day. Charlson Comorbidity Index scores were calculated for all patients, and a multivariable logistic regression was used to calculate odds ratios and confidence intervals. Veterans were included if they had a diabetic foot infection complicated by osteomyelitis, and requiring amputation. Patients were excluded if they had an initial above-the-knee amputation (AKA) or below-the-knee amputation (BKA), since these are generally definitive treatment for osteomyelitis.

**Table 2:**
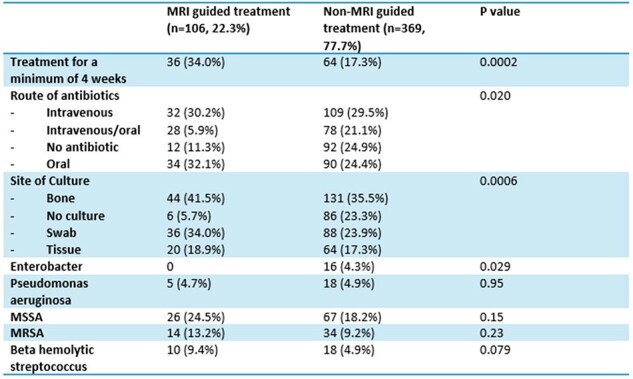
Treatment Characteristics MRI=magnetic resonance imaging, MSSA=methicillin susceptible Staphylococcus aureus, MRSA=methicillin resistant Staphylococcus aureus

**Results:**

There were 1,463 patients based on ICD-10 codes, of which 475 patients underwent amputation. MRI guided therapy occurred in 106 patients. Multivariable logistic regression analysis revealed a 10-fold decrease in the odds of re-amputation within 30 days (OR=0.1; CI 0.01-0.72), a 3-fold decrease within 60 days (OR=0.32; CI 0.13-0.83), and a 2-fold decrease within the 180-day and 365-day (OR=0.51; CI 0.28-0.94 and OR=0.52; CI 0.3-0.9, respectively) intervals when MRI guidance was utilized.

**Table 3:**
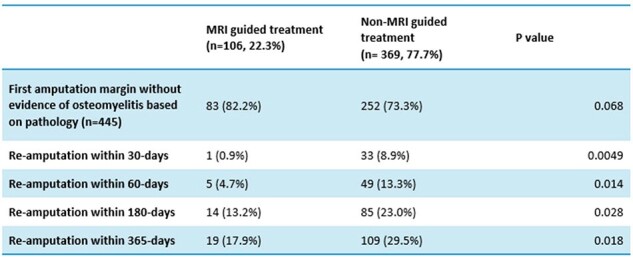
Bivariate analysis of re-amputations MRI=magnetic resonance imaging

**Conclusion:**

These findings suggest that incorporating a pre-amputation MRI into the diagnostic and treatment approach for osteomyelitis associated with diabetic foot infections may reduce the risk for subsequent amputations.

**Table 4:**
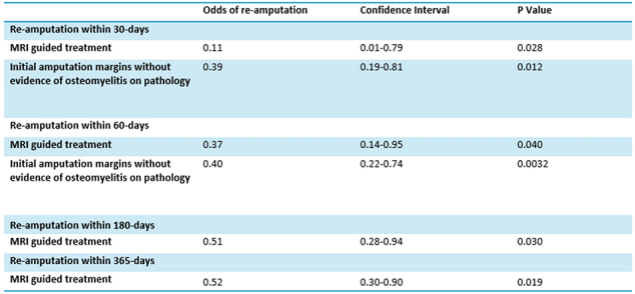
Multivariable analysis of re-amputations MRI= magnetic resonance imaging

**Disclosures:**

**All Authors**: No reported disclosures

